# Supplementary material for: Gender Differences in All-Cause Mortality after Acute Myocardial Infarction: Evidence for a Gender–Age Interaction
Source: J Clin Med. 2022 Jan 21;11(3):541. doi: 10.3390/jcm11030541 (PMC8837133; doi:10.3390/jcm11030541)
Supplement: Supplementary file 1 [file jcm-11-00541-s001.zip › KAMIR-NIH Investigators .pdf]

Acknowledgments

Korean Acute Myocardial Infarction Registry National Institutes of Health (KAMIR-NIH) Investigators: Myung Ho Jeong MD(Chonnam National University Hospital, Gwangju, Republic of Korea, principal investigator, E-mail: myungho@chollian.net), Sung Chull Chae MD(Kyungpook National University Hospital, Daegu, Republic of Korea), Kiyuk Chang MD(Seoul St. Mary's Hospital, College of Medicine, The Catholic University of Korea, Seoul, Republic of Korea), WookSung Chung MD(Seoul St. Mary's Hospital, College of Medicine, The Catholic University of Korea, Seoul, Republic of Korea), TaeHoon Ahn MD(Gachon University, Gil Medical Center, Incheon, Republic of Korea), Seung Woon Rha MD(Korea University, Guro Hospital, Seoul, Republic of Korea), Hyo-Soo Kim MD(Seoul National University Hospital, Seoul, Republic of Korea), HyeonCheol Gwon MD(Sungkyunkwan Universtiy, Samsung Medical Center, Seoul, Republic of Korea), InWhan Seong MD(Chungnam National University Hospital, Daejeon, Republic of Korea), KyungKuk Hwang MD(Chungbuk National University Hospital, Cheongju, Republic of Korea), Kwon-Bae Kim MD(Keimyung University Dongsan Medical Center, Daegu, Republic of Korea), Kwang Soo Cha MD(Pusan National University Hospital, Busan, Republic of Korea), SeokKyu Oh MD(Wonkwang University Hospital, Iksan, Republic of Korea), JeiKeon Chae MD(Chonbuk National University Hospital, Jeonju, Republic of Korea).
