# Supplementary figures and images for: Gender Differences in All-Cause Mortality after Acute Myocardial Infarction: Evidence for a Gender–Age Interaction
Source: J Clin Med. 2022 Jan 21;11(3):541. doi: 10.3390/jcm11030541 (PMC8837133; doi:10.3390/jcm11030541)

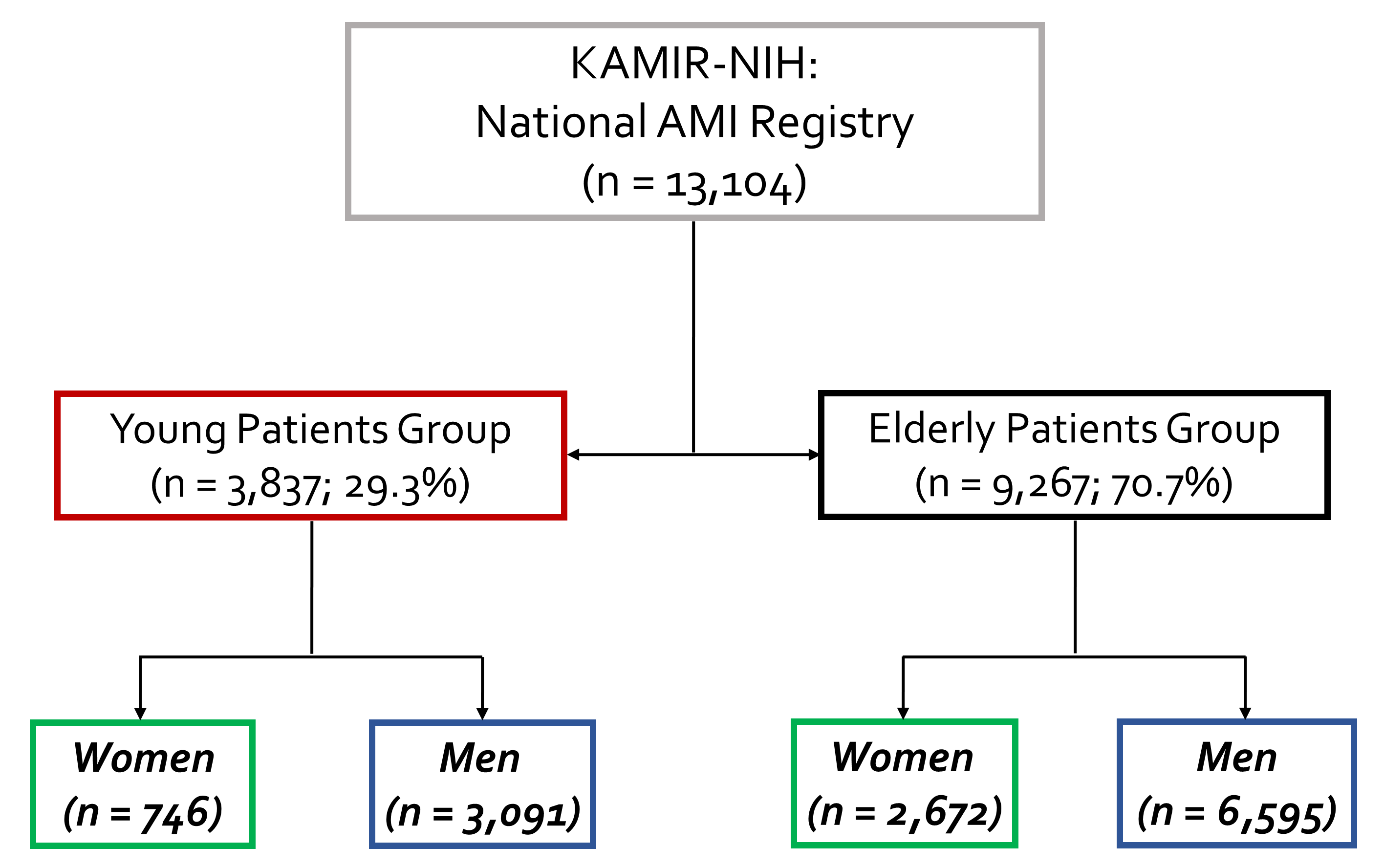

Supplement: Supplementary file 1 [file jcm-11-00541-s001.zip › Figure S1 in the Supplementary Material.tif]

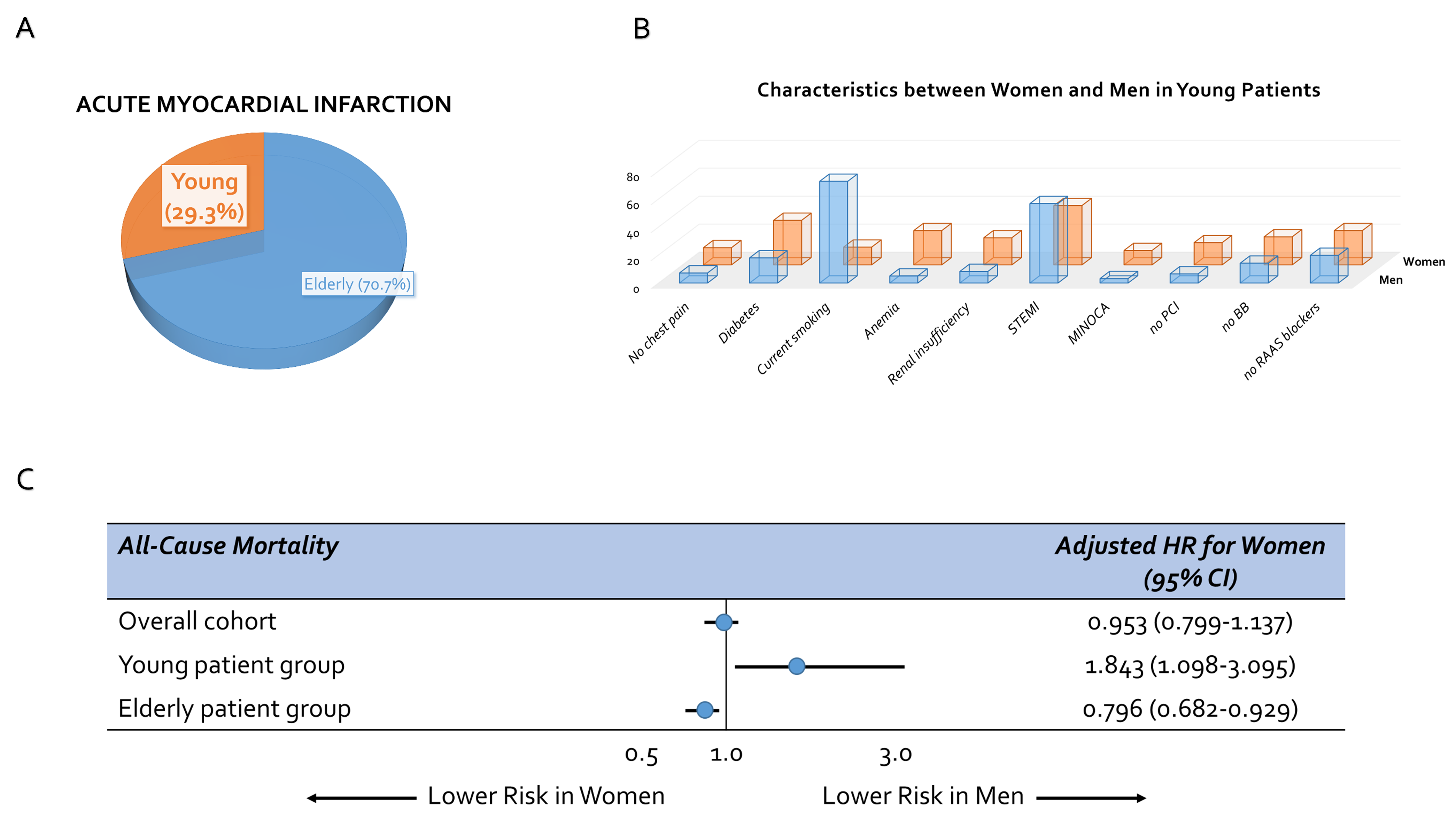

Supplement: Supplementary file 1 [file jcm-11-00541-s001.zip › Figure S2 in the Supplementary Material.tif]
